# Ten simple rules for reporting information on species interactions

**DOI:** 10.1371/journal.pcbi.1010362

**Published:** 2022-08-11

**Authors:** Cristina A. Kita, Guillermo Florez-Montero, Sebastián Montoya-Bustamante, Renata L. Muylaert, Natalya Zapata-Mesa, Marco A. R. Mello

**Affiliations:** 1 Departamento de Ecologia, Instituto de Biociências, Universidade de São Paulo, São Paulo, Brazil; 2 Programa de Pós-Graduação em Ecologia, Instituto de Biociências, Universidade de São Paulo, São Paulo, Brazil; 3 Universidade Federal do ABC, Centro de Ciências Humanas e Naturais, Santo André, Brazil; 4 Molecular Epidemiology and Public Health Laboratory, School of Veterinary Science, Massey University, Palmerston North, New Zealand; Dassault Systemes BIOVIA, UNITED STATES

## Introduction

“*It is like the fire of a torch*: *If hundreds or thousands of people would come each with a torch to ignite by that flame*, *each torch they have ignited from the original one could be used to cook meals and keep a dark house bright*, *and yet the original torch would stay as bright as it used to be*.”—Shakyamuni Buddha, “The Sutra of Forty-two Chapters”

There is growing appreciation of information sharing in science, because it allows reproducibility and boosts usability, thus benefiting the community and helping to advance knowledge [1]. Nevertheless, to be helpful, information sharing needs to be efficient and that depends not only on consistently reporting raw data, but also methods, processed data, and model results. Whenever there are inconsistencies, issues arise. First, issues in reproducibility that arise due to the lack of methodological details reduce science trust. These details are crucial for assessing a study’s reproducibility and reliability [2,3]. Second, issues in the reuse of a study’s raw and processed data that arise due to an incomplete report of them limit their reuse for making synthesis (sensu [4]). An incomplete reporting of model results also hinders synthesis work, which slows down the development of a field.

Aiming to solve those issues and improve the reproducibility and usability of primary scientific research, general guidelines have been proposed in the light of the open science culture [5]. Examples of such guidelines are the FAIR Guiding Principles (FAIR) [6] and the Transparency and Openness Promotion (TOP) [7]. Another outstanding example is the Preferred Reporting Items of Systematic Reviews and Meta-Analyses (PRISMA) [8]. Extensions of those guidelines have also been elaborated to address issues faced in specialized fields. For example, in ecology, our field, there is a new extension known as the Preferred Reporting Items for Systematic Reviews and Meta-Analyses in Ecology and Evolutionary Biology (PRISMA-EcoEvo) [9]. Ecologists also use other specialized guidelines, such as the Tools for Transparency in Ecology and Evolution (TTEE), designed to help journals adopt TOP [10].

Those existent guidelines and extensions are crucial as many ecologists rely on primary data for synthesis. However, despite those new roadmaps and tools, issues in reproducibility and usability are still common in ecological studies. We notice them all the time, as our research group specializes in synthesis. Our main topic of interest is ecological interactions between organisms of different species (a.k.a. species interactions), such as pollination and zoonosis. We have struggled to extract information from primary sources when compiling primary interaction data, conducting meta-analysis, reanalyzing processed data, and interpreting model results. Our syntheses strongly depend on you, who collect data on species interactions in the field or lab. Most importantly, we agree that sharing your data collected with so much effort without receiving proper rewards is not fair [11]. Anyway, despite asymmetric rewards and conflicts of interest, both data producers and users can greatly benefit from an open research culture, as we discuss here.

Aiming to tie those loose ends and improve the communication between data producers and users, and by harnessing the framework created by the previously mentioned guidelines, we propose 10 simple rules for reporting information related to data collection methods, raw data, processed data, and model results from studies on species interactions. Our objective is to go beyond merely pointing out problems, as we also suggest practical solutions to solve them. Although some of our rules apply to researchers who use primary information for secondary studies, they are addressed primarily to you and all colleagues who produce primary information on species interactions. Because our rules can significantly improve the reproducibility and usability of methods, data, and results, by following them you can improve the citations of your primary studies [12] as well as broaden your collaboration and coauthorship horizons. In other words, if you follow our rules, your hard work can benefit the entire scientific community, including your own research group.

### Rule 1: Everything is connected

**Fig 1:**
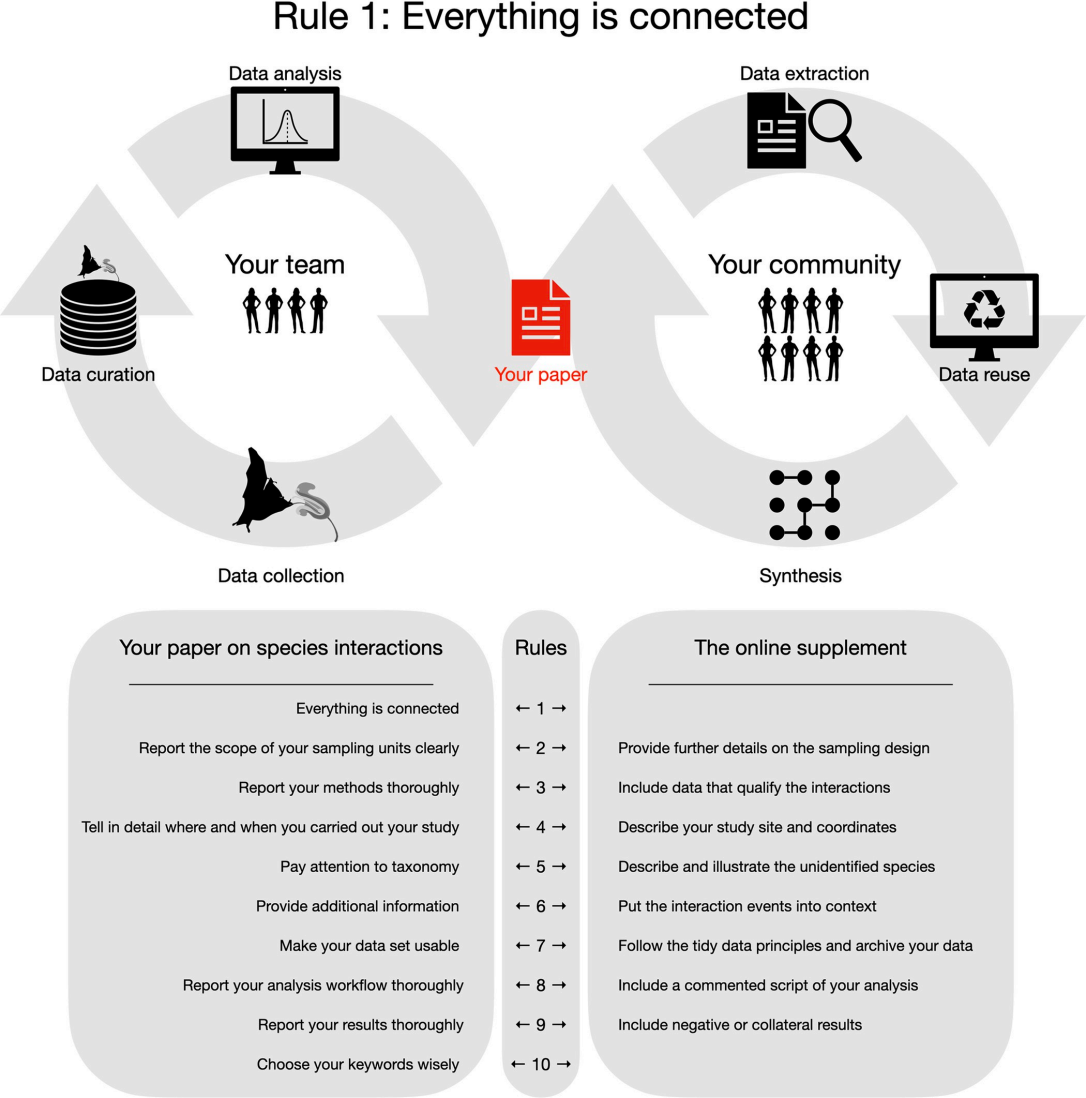
Our 10 simple rules are connected to one another. Follow them and use our roadmap when designing and publishing your study on species interactions. This additional work might boost your study’s impact and usability, thus helping your community and your team. Efficient communication goes a long way.

A good study begins with an exciting problem begging to be solved. From the problem come your questions and expectations, and from them follow the methods used to describe new phenomena or contrast expectations against reality. Adequate methods lead to reliable results, allowing robust interpretations and paving the way for discoveries. Nevertheless, all this fine-tuning might not be helpful for yourself and your community if every step taken along the way is not clearly explained. You cannot bake a tasty cake without a nice recipe. Likewise, the reader cannot assess your study’s reliability and originality if its methods, data, and results are not thoroughly reported. Therefore, if you follow all rules proposed here, the reader will be able to use your ideas (the main goal of any scientific study), reuse your data, and make synthesis with your results. In addition, you can use our rules as guidelines to design your study from scratch, as many people do in the case of systematic reviews and meta-analyses carried out in the light of PRISMA, because all these rules and guidelines are connected to one another (Fig 1).

### Rule 2: Report the scope of your sampling units clearly

We are sure you have also faced this problem when checking a data sheet. Several studies lack clarity about the scope of the sampling units, especially in terms of the ecological level of organization assessed (i.e., individual, population, community, ecosystem, or biome). When samples are taken from wild organisms, some methods capture or collect individuals, while others only allow direct or indirect observation. Those differences also need to be addressed in the statistical analysis. In addition, be clear about recording interactions between individuals or groups of individuals. For example, if samples were taken from individuals that were subsequently released without being marked or collected, they should be quantified as “number of captures” rather than “number of individuals.” If samples were taken from direct or indirect observation, they should be quantified as “number of visits” rather than “number of individuals.” These simple changes can improve statistical analysis and communication with your peers.

### Rule 3: Report your methods thoroughly

Not all primary studies allow us to reproduce their data collection unequivocally. Naive assumptions must be made when we lack information about study design, which leads to biases [13]. Therefore, provide all details needed to calculate the sampling effort following the standards accepted in your field. Provide rich information about capturing and recording devices (manufacturer, model, size, and material), hours sampled per field session, number of sampling sessions per time unit (e.g., day, week, month, season, or year), and number and size of sampling plots within sampling sites. If multiple sites have been sampled, detailed information should be reported for each one.

In addition to being clear about the sampling sites, informing the size of the team of data collectors, as well as how the team was divided across sites and treatments, can help the readers to understand sampling effort and potential biases. Understanding those key points can prevent serious biases as, for example, the number of nodes and links in an ecological network tends to increase with sampling effort. This trend can induce a potential bias toward a core of highly connected species, underestimating the presence of lowly connected species, which results in flawed assessments of network topology [14,15]. Diagrams are particularly welcome to explain your sampling design. It is also essential to state the limitations of your methods, for example, in terms of the taxonomic groups included or excluded.

Finally, report interaction attributes that allow telling apart positive and negative interaction events (e.g., the reproductive organs of a flower were touched or not by the visitor?) and explicitly state if your data set has only positive, negative, or both types of interactions. This way, you can avoid misinterpretations and clarify potential limitations of your data set. Moreover, remember to explain what the interaction weights mean in your study (e.g., frequency of encounter or number of resources harvested?). Due to the difficulty in comparing interaction strength (measured as, for example, the sum of dependencies of a species [16]) between different interaction types, we also recommend reporting raw data on species abundance and number of interaction events.

### Rule 4: Tell in detail where and when you carried out your study

Unfortunately, critical information about your study area might go unnoticed, despite being essential to make your paper reproducible and usable. Therefore, describing the study area in detail is particularly important when analyzing spatial and temporal patterns of species interactions. Whenever possible, take a professional GPS to the field so that you can georeference your sampling sites, plots, transects, and trails. Afterwards, it is worth reporting all coordinates in decimal degrees using the proper datum, preferably the World Geodetic System 1984 (datum WGS 84). If you do not have a professional GPS, download a geotracking app to your smartphone, as modern models contain GPS receivers with good accuracy in open areas. You can also use indirect approaches, such as identifying site landmarks on Google Maps or Google Earth and extracting their coordinates. Explain the georeferencing in detail as each technique implicates different accuracy levels.

When reporting the seasons sampled, consider universal seasons, such as summer/winter and spring/fall, and local seasons that affect the interactions studied, such as rainy/dry. You can also report microclimatic information, such as temperature, moisture, and rainfall. Next, illustrate the environment, describe its elevation, types of vegetation, average tree height, water sources, and any other information that helps assess the context of the interactions. Finally, explain the land-use regimes of the studied landscape. If possible, include a map in the supplement.

### Rule 5: Pay attention to taxonomy

Identifying organisms to the species is no easy task. In addition, many studies do not name species correctly, with mistakes varying from misspelling to outdated names. Those 2 problems combined create severe limitations for interpreting and reusing interaction data. Scientific names are not a mere formality, but they are key to unlocking a trove of biological knowledge acquired over generations. Access to this knowledge is crucial to correctly interpret the conditions and resources required by the species involved in the interactions. Furthermore, this knowledge is crucial to tell apart closely related types of interactions, such as seed dispersal and seed destruction, based on their potential outcome [17].

Therefore, whenever possible, provide additional information about unidentified species, such as photos, sketches, DNA/RNA sequences, and ultrasound calls, in the supplement so that other scientists can at least tell different morphospecies apart. Thus, when dealing with unidentified species, rather than grouping them all together per higher taxon, it is better to number them individually for each study site. Remember that morphospecies 1 found at site A is not necessarily the same as morphospecies 1 found at site B, especially if the sites are far away from one another.

Most importantly, name the species correctly, following international taxonomic standards [18,19], including correct spelling and up-to-date names recognized for the taxon. There are many publicly available taxonomic databases that can help you, some of them focused on a single taxonomic group such as Mammal Diversity Database (https://www.mammaldiversity.org/) and Plants of the World Online (http://powo.science.kew.org/), and others with broad taxonomic scope such as Catalogue of Life (https://www.catalogueoflife.org/) and Encyclopedia of Life (https://eol.org/). There are also some awesome tools for taxonomic harmonization [20].

Some very helpful packages for R [21] are also available. For example, packages that allow users to download phylogenic and taxonomic data directly in R such as *rotl* [22], packages for parsing, plotting, and manipulating large taxonomic data sets such as *metacoder* [23], and even brand new packages for checking taxonomic spelling such as *taxspell* (https://github.com/sckott/taxspell). Reporting the taxonomy reference used is also a good practice that improves communication with your peers. Remember that taxonomy changes over time, so names are vital in connecting knowledge from studies separated by decades or centuries.

Finally, when publishing the data, do not only present species codes but also write full scientific names in a data frame used as a species reference in the supplement. Double check that no species code is left without its corresponding full name. And always invite as a coauthor a specialized taxonomist, who can check the names in your database and connect the dots in the literature. There is no substitute for expert knowledge and experience.

### Rule 6: Provide additional information

You should always record and report additional information not considered in the original data collection plan. An excellent way to do that is field notes, which shed light on potential sources of bias, such as rain, cloudy days, fires, floods, hurricanes, earthquakes, volcano eruptions, or other outstanding events. This kind of information puts the data into context and helps other researchers interpret outliers and formulate new questions. OK, we know that the current publication ethos pushes us toward being extremely concise in our articles, but we can make unlimited use of online supplements to tell richer stories. For example, if a bat captured on a given night had an abnormal amount of ectoparasitic flies for its species, that is undoubtedly worth mentioning. Reporting additional information was common practice in the time of classical naturalists and has always helped people think outside the box. Just remember Alexander von Humboldt and his marvelous field notes [24].

### Rule 7: Make your data set usable

Sharing is caring, so mind the data you share. You should prepare your primary data to be readily used in reanalysis, new analysis, and synthesis [25]. This care may open many new research avenues and boost interest in your work. Unfortunately, many studies report data as tables embedded in the text, usually in PDF format, which seriously hinders manual and automatized data extraction. In addition, typing data from PDFs also increases the chances for errors. Instead, raw data should be shared in data sheets in plain text formats, such as TXT or CSV, which any software running on any operating system can process.

Likewise, you should follow a tidy data format to organize your sheets as it creates human- and machine-readable, easily manipulated data. The principles of tidy data provide a standard way to organize data values within a data set and are pretty simple [26]: (1) Each variable forms a column; (2) each observation forms a row; and (3) each type of observational unit forms a table. For example, if you captured bat species A, carrying seeds of plant species X at 19:00 and plant species Y at 20:00, in the mist nets α and β, respectively, the columns of your data table could be named “bat species,” “plant species,” “hour,” and “mist net ID.” Then, the first row would read “A; X; 19; α” and the second, “A; Y; 20; β.” If some information is missing, you can fill a cell with “not available” (i.e., NA).

Once you have a tidy data set, you can use tidy tools for data analysis, in which the output of one tool can be used as the input of another. This allows you to combine multiple tools to solve a complex problem in a reliable and reproducible way. In addition, remember to create a metadata file that explains the content of each table and column, as well as the codes used to summarize information. Many good guides help you accomplish this task [27,28]. Yes, we know that this is much information to include in a manuscript. Therefore, move data and metadata to the supplement or, even better, to open databases and repositories (adequately cited in the manuscript using stable URLs). See more tips for data archiving in the next rules.

### Rule 8: Report your analysis workflow thoroughly

Have you ever had trouble understanding and reproducing the analysis workflow of a particular study or even of a study you carried out years ago? Nowadays, large amounts of data in ecology are analyzed by coding, using languages such as R, Python, MATLAB, C++, or Julia. Sadly, code is not shared in most studies [29]. Therefore, the best solution for this problem is to provide a script file with the code used in your analysis, complemented by the processed data that you directly used in the analysis. For example, instead of only reporting network metrics (e.g., nestedness, connectance, and modularity), report also the language (e.g., R or Python), the package (e.g., *bipartite* or *igraph*), and the function (e.g., *computeModules or cluster_louvain*) you used. You can even go beyond sharing code by writing tutorials in Markdown, LaTeX, and other languages, which guide the reader when reproducing your analysis. If you did not analyze your data by coding, provide a step-by-step textual description (i.e., pseudocode) that describes how you got your results. This enables full reproducibility.

After preparing the supplement carefully, we strongly recommend that you deposit your code and data in an online public repository. This is already a common practice with genetic data put on GenBank (http://www.ncbi.nlm.nih.gov/genbank/) and with animal tracking data that go to MoveBank (https://www.movebank.org/). In our field, you can deposit species interaction data on GloBI (https://www.globalbioticinteractions.org/data), Mangal (https://mangal.io/), Lifewebs (http://www.lifewebs.net/), or Web of Life (https://www.web-of-life.es), among other repositories. If you work with vertebrate-virus associations, you can deposit your data on the VIRION database (https://www.viralemergence.org/data). There are also more general open repositories such as Zenodo (https://zenodo.org) and Dryad (https://datadryad.org), which allow creating stable URLs and citable DOIs for GitHub repositories, help you choose licenses, and provide long-term archiving [25].

Archiving is a practice that ecology and evolutionary biology journals and funding agencies have been encouraging or requiring [30]. By doing this, you contribute to making your data accessible and reusable in a transparent way. Moreover, you can broaden your coauthorship horizons. For example, the Lifewebs repository (http://www.lifewebs.net/contribute.html) offers to all data contributors authorship in resulting publications in which their data sets were used. In other words, by archiving your data in an online public repository, you can increase not only the citation of your primary studies but also the number of your publications.

Do not worry about making your data publicly available, as when archiving code and data you can inhibit unwanted manners of use by choosing an adequate license. There are 6 license options on Creative Commons (a.k.a. CC licenses; https://creativecommons.org/), ranging from most to least permissive. The most permissive is CC BY, which allows users to distribute, remix, adapt, and build upon the material in any medium or format, so long as attribution is given to the creator. This license also allows commercial use. The least permissive is CC BY-NC-ND, which allows users to copy and distribute the material in any medium or format in unadapted form only, for noncommercial purposes only, and only so long as attribution is given to the creator. There is also an option called CC0 (a.k.a. CC Zero) that is a public domain dedication tool that allows users to distribute, remix, adapt, and build upon the material in any medium or format, with no conditions. Depending on the repository used, you can choose any of the standard licenses included in the tools.

In addition to repositories and licenses, there are some guidelines available on blogs and other publications that you can follow to improve your script and make it more useful (e.g., [29,31]). This way, readers and users will find all the answers they need, making your analysis workflow easily reproducible. Besides reproducibility, providing your code as a script has other benefits, such as making your results checkable and reliable, which may improve your study’s impact. This practice may also be pedagogical for young scientists, which contributes to the open science culture.

### Rule 9: Report your results thoroughly

Do not spare any details when reporting your model results after all this work to make your study reproducible and reusable. Many ecological studies report only *P* values or cherry-pick the results that support the working hypothesis [32]. Missing results, negative or positive, hamper data extraction and significantly affect reliability and usability. For example, uncertainty concerning a subset of results can bias a meta-analysis, as excluding studies with missing information worsens the publication bias [33]. Biases of different kinds can lead to worrisome practical consequences, such as not detecting the harmful effect of a pesticide on crop pollination.

In addition, fraud is more easily prevented by transparency. When reporting results from tests or models, go beyond significance values by including relevant descriptive statistics, scores of the calculated statistics, sample sizes, degrees of freedom, effect sizes, and statistical powers (e.g., [34]). If a result does not belong to the core of the story being told, but is important to help understand its context, move it to the supplement. When reporting results in figures, use transparency to indicate data overlap and incorporate measures of variability (such as variance, standard deviation, or standard error) in the figure or its caption [29].

### Rule 10: Choose your keywords wisely

This last rule might sound trivial but pay close attention to it. All your hard work is lost if people do not find your paper, so choose your keywords wisely. Keywords not only help people interested in the same scientific problem find your paper, but they are also crucial for people who carry out systematic reviews, meta-analyses, and all kinds of synthesis. Unfortunately, several studies on species interactions are overlooked in advanced searches due to poor keyword choice.

Therefore, first, we strongly recommend that you include at least one of the following general keywords, even if they are already contained in the title: “ecological associations,” “interspecific interactions,” or “species interactions.” Second, add keywords related to the expected outcome and intimacy of the interactions, such as “amensalism,” “antagonism,” “commensalism,” “mutualism,” or “symbiosis.” Third, include some keywords specific to the studied interaction type, such as “blood parasitism,” “cleaning symbiosis,” “ectoparasitism,” “endoparasitism,” “extrafloral nectaries,” “folivory,” “florivory,” “frugivory,” “infection,” “nectarivory,” “nectar robbery,” “oil collection,” “pollination,” “pollinivory,” “seed dispersal,” “trophobiosis,” “zoonosis,” or whatever fits your study best.

By choosing your keywords wisely, your work will gain visibility and will more likely be found, read, cited, and used in synthesis.

### Final remarks

The reproducibility crisis in global science is also worrisome in the small world of species interactions. Transparency and clarity are crucial to solving this crisis. Furthermore, scientists who collect primary interaction data in the field or lab can significantly benefit from improving the reproducibility and usability of their studies. Like a torch, whose brightness is not diminished by igniting other torches, the reuse of primary data and the synthesis of primary results broaden the scope of your primary studies by multiplying their potential uses, boosting their citations, and creating new opportunities for collaboration and coauthorship. To achieve this promising scenario of mutual benefits, improving communication between data producers and users is of paramount importance. The 10 simple rules suggested here can help us reach this goal.
